# Freshwater species distributions along thermal gradients

**DOI:** 10.1002/ece3.4659

**Published:** 2018-12-18

**Authors:** Oskar Kärcher, Daniel Hering, Karin Frank, Danijela Markovic

**Affiliations:** ^1^ Faculty of Business Management and Social Sciences Osnabrück University of Applied Sciences Osnabrück Germany; ^2^ Faculty of Biology, Aquatic Ecology University of Duisburg‐Essen Essen Germany; ^3^ UFZ – Helmholtz Centre for Environmental Research Ltd Department for Ecological Modelling Leipzig Germany; ^4^ Institute of Environmental Systems Research University of Osnabrück Osnabrück Germany; ^5^ iDiv – German Centre for Integrative Biodiversity Research Halle‐Jena‐Leipzig Leipzig Germany

**Keywords:** climate change, European freshwater, generalized additive models, preferred temperature, safety margin, thermal response, warming tolerance

## Abstract

The distribution of a species along a thermal gradient is commonly approximated by a unimodal response curve, with a characteristic single optimum near the temperature where a species is most likely to be found, and a decreasing probability of occurrence away from the optimum. We aimed at identifying thermal response curves (TRCs) of European freshwater species and evaluating the potential impact of climate warming across species, taxonomic groups, and latitude. We first applied generalized additive models using catchment‐scale global data on distribution ranges of 577 freshwater species native to Europe and four different temperature variables (the current annual mean air/water temperature and the maximum air/water temperature of the warmest month) to describe species TRCs. We then classified TRCs into one of eight curve types and identified spatial patterns in thermal responses. Finally, we integrated empirical TRCs and the projected geographic distribution of climate warming to evaluate the effect of rising temperatures on species’ distributions. For the different temperature variables, 390–463 of 577 species (67.6%–80.2%) were characterized by a unimodal TRC. The number of species with a unimodal TRC decreased from central toward northern and southern Europe. Warming tolerance (WT = maximum temperature of occurrence—preferred temperature) was higher at higher latitudes. Preferred temperature of many species is already exceeded. Rising temperatures will affect most Mediterranean species. We demonstrated that freshwater species’ occurrence probabilities are most frequently unimodal. The impact of the global climate warming on species distributions is species and latitude dependent. Among the studied taxonomic groups, rising temperatures will be most detrimental to fish. Our findings support the efforts of catchment‐based freshwater management and conservation in the face of global warming.

## 1 INTRODUCTION

Freshwater ecosystems cover <1% of the Earth's surface, but are home to around 6% of all known species (Strayer & Dudgeon, 2010). The warming rates of recent decades combined with the multitude of anthropogenic stressors threaten the biological diversity, structure, and function of freshwater ecosystems (Mantyka‐Pringle, Martin, Moffatt, Linke, & Rhodes, 2014; Strayer & Dudgeon, 2010; Woodward, Perkins, & Brown, 2010). Habitat fragmentation and the limited ability of many species to track spatial shifts toward suitable habitats cause freshwater biodiversity to be highly vulnerable to climate warming (cf. Markovic, Carrizo, Kärcher, Walz, & David, 2017).

The magnitude of the already observed temperature alterations plays a fundamental role for determining the future climatic suitability of current species’ ranges. Temperature has strong impacts on the physiology (Vornanen, Haverinen, & Egginton, 2014), growth (Elliott & Allonby, 2013) and behavior of certain species (Frost et al., 2013). According to the Fifth Assessment Report of the Intergovernmental Panel on Climate Change (IPCC, 2013), the linear trend of the globally averaged combined land and ocean surface temperature data show a warming of 0.85°C (0.65–1.06°C), over the period 1880–2012 (IPCC, 2013). Recent warming was shown to drastically shift the ranges of different taxonomic groups (Chen, Hill, Ohlemueller, Roy, & Thomas, 2011; Domisch et al., 2013; Markovic et al., 2017), leading to a decline of many populations (Parmesan, 2006). In particular, for strictly aquatic species, temperature may set environmental tolerance range limits (Wiens, 2011). Accordingly, already minor shifts in water temperature lead to considerable changes of species assemblages.

The typical assumption for a thermal response curve of a species is the Gaussian curve (Gauch & Whittaker, 1972), with the preferred temperature at its peak. For freshwater species, responses along thermal gradients are sparsely explored, providing the opportunity to investigate current thermal response shapes. Most previous studies on the thermal responses of freshwater species have been constrained to single taxonomic groups or to single stream networks. For example, using the logistic generalized linear regression models (GLMs), Logez, Bady, and Pont (2012) have identified thermal responses for 21 native European fish species, while Isaak, Wenger, and Young (2017) identified thermal responses for 14 fish and amphibian species for a mountain stream network in the U.S. Rocky Mountains. Similarly, Pyne and Poff (2017) identified insect taxon response curves for temperature and streamflow. Comparative studies delineating response curves of species from various taxonomic groups are missing.

Assessing species responses along environmental gradients commonly involves the use of various statistical approaches that estimate the probability of a species’ occurrence as a function of the environmental conditions across the current species’ geographic range, that is, the environmental response curves. Generalized linear regression models are among the most widely used approaches for identifying response curves. However, there are numerous alternative approaches such as 95%‐quantile regressions (Carrascal, Villén‐Pérez, & Palomino, 2016) or Huisman‐Olff‐Fresco models (HOF) (Huisman, Olff, & Fresco, 1993). The latter are considered one of the best statistical tools for response modeling, because of their predictive performance (Jansen & Oksanen, 2013; Oksanen & Minchin, 2002). It was shown that HOF models perform better than GLMs or beta functions (Lawesson, Fosaa, & Olsen, 2003; Oksanen & Minchin, 2002). Generalized additive models (GAMs) provide response curves that coincide with the shape of those resulting from the HOF models (Jansen & Oksanen, 2013). Specifically, Jansen and Oksanen (2013) have shown that the HOF models were mostly located in the range of the 95% confidence interval of GAMs. Additionally, according to Oksanen and Minchin (2002), GAMs and HOF models usually were consistent, but GAM has a greater flexibility regarding the response shape than HOF models, which are restricted to a limited number of shapes.

This study explores and compares the thermal responses of 577 European freshwater species of molluscs, fish, plants, odonates, and crayfish. Thermal properties derived from global species ranges (209,659 catchments) are transferred to the European scale (16,689 catchments). We use GAMs to link the species occurrence data to the annual mean air/water temperature and to the maximum air/water temperature of the warmest month, respectively, to parameterize species’ thermal response curves (TRCs). Specifically, we examine and compare the TRC types for the different temperature variables and the thermal properties across the individual species, taxa groups, and latitudes. The TRCs link the species occurrence probability to temperature patterns and are thus of fundamental importance for the conservation of freshwater biodiversity given the current warming rates and the likelihood of further temperature increases (cf. Isaak et al., 2017). Finally, we match the empirical thermal response curves with the projected temperature for the middle of the 21st century to evaluate the impacts of temperature alterations on freshwater species distributions throughout Europe.

## 2 METHODS

### 2.1 Species data

The IUCN Global Species Programme, as part of the Red List assessment process (IUCN, 2013, 2014), compiled presence and absence data on freshwater species distribution ranges in polygon shape files corresponding to global watershed boundaries. To capture the whole range of freshwater species native to Europe, the global species data from the IUCN Global Species Programme were used. Global data were available for 1,402 freshwater species native to Europe including 609 molluscs, 473 fishes, 209 plants, 106 odonates, and five crayfish (see https://www.iucn.org/theme/species/our-work/iucn-red-list-threatened-species for more details). Freshwater species data were mapped to 209,659 catchments at the HydroBasins level 8 resolution (Lehner & Grill, 2013) (see Supporting Information, Appendix S1, Figure S1.1). Only species that occurred in at least 50 catchments were part of the analysis to guarantee an accurate estimate of the TRCs (Coudon & Gégout, 2007). Due to the dendritic structure of river networks, catchment mapping is more appropriate for freshwater species than the point‐to‐grid mapping used for mapping terrestrial species’ occurrences (see Fagan, 2002). In addition, given that catchments serve as units for freshwater management and conservation (commonly referred to as the Catchment‐Based Approach—CaBA, see DEFRA, 2013), catchment‐scale mapping of freshwater species’ occurrences ensures compatibility between the management and the analysis scales (Lévêque, Oberdorff, Paugy, Stiassny, & Tedesco, 2008; Markovic et al., 2017).

### 2.2 Climate data

Global climatic data were ascertained for the second half of the 20th century (1960–1990, hereafter referred to as baseline) from the WorldClim (version 1.4) 30 arc‐second (approximately 1 km × 1 km) dataset (Hijmans, Cameron, Parra, Jones, & Jarvis, 2005, www.worldclim.org, accessed on March 19, 2018). Due to a lack of in situ and satellite‐retrieved water temperature data given the large spatial extent of our study (209,659 river catchments), parameterization of species’ thermal response curves was based on the catchment‐specific annual mean air temperature (Tmean_air_) and the maximum air temperature of the warmest month (Tmax_air_) of the baseline period. However, given a strong correlation between water and air temperature (Markovic, Scharfenberger, Schmutz, Pletterbauer, & Wolter, 2013; Mohseni, Stefan, & Eriksson, 1998), we used a global relationship model to transform air temperature to stream water temperature on a monthly basis (Punzet, Voß, Voß, Kynast, & Bärlund, 2012). Thus, we estimated the annual mean water temperature (Tmean_water_) and the maximum water temperature of the warmest month (Tmax_water_). The annual mean water temperature was derived by averaging the transformed monthly average air temperatures. Areas without appreciable flows, that is, lakes, reservoirs, and lagoons, were excluded from the analysis. Pairwise Pearson's correlations among the four used variables ranged from 0.81 to 0.98 (Supporting Information Table S1.1).

Future climate projections for Europe (16,689 river catchments) were gathered for the middle of the 21st century (hereafter referred to as 2050s) from the CIAT (International Center for Tropical Agriculture) 30 arc‐seconds gridded dataset (www.ccafs-climate.org). The projections in the CIAT dataset were obtained by three climate models (MOHC, IPSL, and MPI), each considering the RCP4.5 (Representative Concentration Pathways) emission scenario. RCP4.5 follows a medium‐low mitigation of greenhouse gas emission and represents intermediate scenarios (van Vuuren et al., 2011). The gridded layers of the 20th and 21st century Tmean_air_ and Tmax_air_ were mapped to HydroBasins level 8 resolution catchments using the ESRI ArcGIS zonal statistics tool and afterwards transformed to projections of Tmean_water_ and Tmax_water_ using the derived global relationships model (Punzet et al., 2012).

### 2.3 Modeling thermal response curves

#### 2.3.1 Statistical model

Global distributions of freshwater species native to Europe were modeled using GAMs (Hastie, 2016). GAMs are useful to model nonlinear relationships and for relating binary data to probabilities by an adequate transformation of the fit. The evaluation of the species’ thermal response curves for the four different temperature variables Tmean_air_, Tmax_air_, Tmean_water_, and Tmax_water_ (four models per species) was based on a univariate modeling approach, that is, Tmean_air_, Tmax_air_, Tmean_water_, or Tmax_water_ was the only explanatory variable, respectively. Furthermore, a smoothing by spline functions with three degrees of freedom, that is, a piecewise interpolation by polynomials of maximal order two, was applied in order to get a smooth representation of the probability.

Based on the probability results from the statistical model, a threshold for separating presences and absences of a species was determined by minimizing the absolute difference between specificity (the rate of correctly predicted absences) and sensitivity (the rate of correctly predicted presences) (Fielding & Bell, 1997). Minimizing the difference between the sensitivity and specificity generally leads to accurate predictions (Jimenez‐Valverde & Lobo, 2007).

To evaluate the models’ performance, two main measures were calculated: the area under the receiver operating characteristic (ROC) curve, AUC (Hosmer & Lemeshow, 2000), and the true skill statistic (TSS = sensitivity + specificity – 1), whereas specificity and sensitivity are the result of the probability threshold determination (Allouche, Tsoar, & Kadmon, 2006). AUC values can range from 0 to 1, with values of 0.5–0.7 demonstrating poor performance, 0.7–0.9 moderate, and >0.9 high performance (Manel, Williams, & Ormerod, 2001; Swets, 1988). An AUC value of 0.5 indicates a random prediction while an AUC value of 0 means that every presence is incorrectly predicted. TSS values range from −1 to +1, where values ≤0 indicate a random and +1 a perfect performance (Allouche et al., 2006). Consequently, only species with thermal modeling results fulfilling AUC ≥0.7 and TSS ≥0.4 for all four temperature variables were included in further investigations.

To account for accuracy of the predictive performance, the data were split into a training (80%) and validation (20%) dataset. The random data splitting into the training and the validation datasets procedure was repeated 100 times, leading to 100 individual values of the main performance measures for the calibration and validation phase, respectively, which were averaged afterwards (Dormann, Purschke, Márquez, Lautenbach, & Schröder, 2008). The average AUC and TSS values of the validation were used for the assessment of the predictive performance.

Uncertainty was depicted by calculating 95% confidence intervals (CIs) around the modeled probabilities of occurrence, that is, around the thermal response curves, for each observation. CIs give an impression of the scattering and the preciseness of statistically calculated key figures (De Jong & Heller, 2008).

#### 2.3.2 Thermal response curve types

The resulting thermal response curves for each of the four temperature variables, illustrating the probability of occurrence along the thermal gradient, were classified into eight different curve types (see Table 2). Type I corresponds to a Gaussian distribution, that is, a unimodal symmetric response, showing a uniform distribution of the species’ occurrence around the temperature with the highest probability of occurrence (here termed as “preferred temperature”). Type II represents a unimodal right skewed response and thus a tendency toward warmer regions. Type III describes a unimodal left skewed response, representing the tendency toward colder regions, that is, regions below the preferred temperature. Type IV represents no response, that is, the response curve is approximately a constant line at some probability. Type V describes an increasing probability of occurrence up to a certain threshold and an afterwards nearly constant response at the height of the respective threshold, showing a constant probability for higher temperatures. Type VI corresponds to a mirror image of the Type V response. Type VII response is characterized by a monotonic growth and Type VIII by a monotonic decline, indicating higher or lower probabilities along cold to warm temperatures, respectively (see Table 2).

Responses were automatically identified as Type IV, that is, no response, if the maximum probability of occurrence was smaller than 0.01, because for these low probability TRCs no reliability can be assumed. In cases of a maximum probability greater than or equal to 0.01, all types were taken into consideration in order to determine the type via an automatic identification that makes use of the slope properties of each thermal response curve.

#### 2.3.3 Assessment of species’ thermal properties

The global thermal range of each single species is defined as the current temperature range. Thus, the thermal range is the difference between the maximum temperature and the minimum temperature of occurrence. Thermal ranges or breadths facilitate the understanding of the vulnerability to extinction and of the rarity of a species (Slatyer, Hirst, & Sexton, 2013). Additionally, for each of the four statistical models, a thermal preference for species of Types I‐III, that is, for species with unimodal responses, was specified. The preferred temperature (*T*_pref_) is the temperature with the highest probability of occurrence. *T*_pref_ was determined by using the function “optimize” implemented in R (R Development Core Team, 2017), which searches for the maximum probability. The maximum temperature at which the species was registered for each temperature variable was set as critical temperature (CT).

Species sensitivity to global warming is closely related to species’ thermal range, thermal distribution and preferred temperature (cf. Markovic et al., 2017). For example, species with a small thermal range and low CT are more likely to be sensitive to rising temperatures. The potential exposure to global warming at the European scale was quantified using the difference between the average of the respective projected temperature variables of the three climate models and the corresponding species‐specific CT. The difference was considered “critical” if the projected temperature exceeded CT (i.e., the current baseline maximum temperature of occurrence of the species). “Warming tolerance” (WT) was calculated as the difference between CT and T_pref_ of the statistical model (WT =CT – T_pref_). For each temperature variable, “safety margin” (SM) was calculated as the difference between T_pref_ and the average temperature of the species’ current temperature range (T_av_) (SM =T_pref_ – T_av_). WT and SM values were derivable only for species with a unimodal response curve (Types I‐III). Geographical variations at the European scale of these tolerance measures were depicted by averaging across latitude. We note that the critical temperature (CT), safety margin (SM), and the warming tolerance (WT) are the common terms used to describe the species thermal performance curves (TPCs) (see Deutsch et al., 2008). Here, we used the latter terms to provide comparable descriptors of the TRCs, but underline that the interpretation of the CT, SM, and WT in the context of TRCs and TPCs is different. Specifically, while TPCs address the question of the species’ performance within a certain thermal range, the TRCs address the question of the likelihood of species occurrence.

## 3 RESULTS

Results from transformed temperature variables, that is, annual mean water temperature and maximum water temperature of the warmest month, led to similar patterns in thermal response curves and thermal properties of the considered species. Therefore, the focus of the results and the following discussion will be on the non‐transformed temperature variables, that is, annual mean air temperature and maximum air temperature of the warmest month. However, results for the water temperature variables are presented in the Supporting Information Appendix S1 (Table S1.2, Figures S1.7 – S1.16) and S2 (Tables S2.3 and S2.4).

### 3.1 Species thermal range

**Figure 1:**
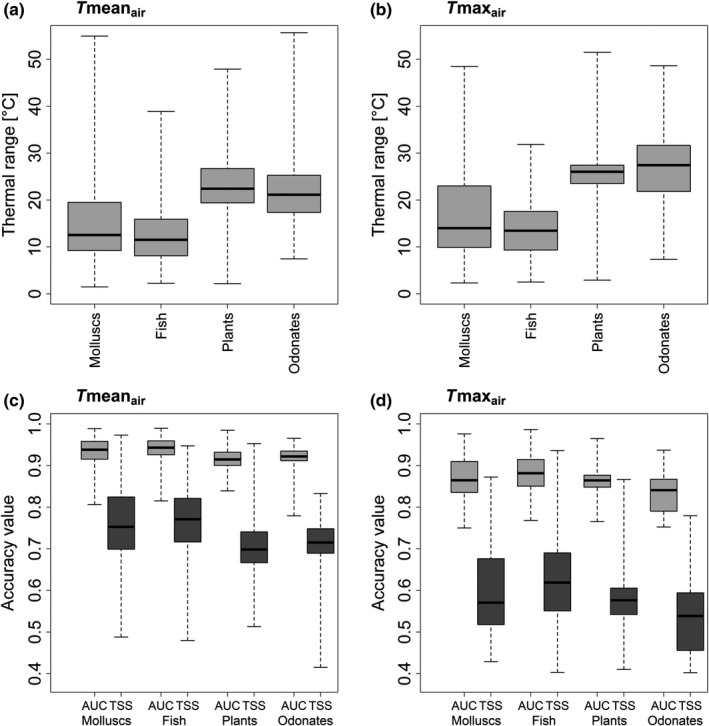
Thermal ranges of the species and the distribution of the accuracy measures per taxonomic group for the respective temperature variable, that is, for (a, c) Tmean_air_ and (b, d) *T*max_air_. The boxplots illustrate the distribution of the minimum, 25% quantile, median, 75% quantile, and maximum of the thermal ranges. The minimum and maximum are displayed by the end of the corresponding whiskers. Note that crayfish were excluded because of the low frequency of analyzed species

The current thermal ranges based on the global species ranges varied greatly across the taxa groups (see Figure 1a,b, Supporting Information Tables S2.1 and S2.2; for convenience, in figures and tables taxa groups are ordered according to the number of initially available species). The globe wanderer (*Pantala flavescens*), the freshwater snail big‐ear radix (*Radix auricularia*), the widely distributed pea clam (*Pisidium casertanum*), and the water‐starwort (*Callitriche brutia*) with thermal ranges above 47°C and 45°C for Tmean_air_ and Tmax_air_, respectively, were among the species with the highest thermal ranges. The molluscs species *Turricaspia lindholmiana*, restricted to the estuarine waters of the Dnieper River system (Ukraine) and the Don River system (Russia), had the smallest realized thermal range regarding both air temperature variables (1.5°C for Tmean_air_ and 2.3°C for Tmax_air_). While for Tmean_air_the second smallest realized thermal range was assigned to the fern *Marsilea batardae*(2.2°C) endemic to the Iberian Peninsula, for Tmax_air_ the fish species *Percarina maeotica* had the second smallest thermal range for Tmax_air_ (2.5°C). The median of the realized thermal ranges was smallest for fish and molluscs (Figure 1a,b).

### 3.2 Models’ performance and uncertainty

Of the initially 1,402 considered European freshwater species, 649 species occurred in more than 50 catchments and were thus suitable for the species distribution modeling. Of the 649 species whose spatial distributions were modeled using GAM, validation model performance was moderate to high (0.7 ≤ AUC ≤1 and 0.4 ≤ TSS ≤1) across the temperature variables for 577 species (see Table 1, Figure 1c,d, Supporting Information Tables S2.1 and S2.2). Models with AUC <0.7 and TSS <0.4 were considered insufficiently accurate, which led to an elimination of the corresponding species from the further analysis (*n* = 72). The validation AUC and TSS values were highest for fish (0.94 ≥ AUC median ≥0.88 and 0.77 ≥ TSS median ≥0.61) and lowest for plants and odonates (0.92 ≥ AUC median ≥0.84 and 0.71 ≥ TSS median ≥0.54). The uncertainty of the modeled occurrence probabilities was low.

**Table 1:** Development of the species number per taxonomic group. The table includes the initial number of species, the number of species, which occurred in at least 50 catchments, and the number of species, which fulfilled the statistical model accuracy criteria for all four temperature variables, that is, the AUC and TSS values of the species’ statistical thermal response curve model were >0.7 and 0.4, respectively

Taxonomic group	No. species	No. species with *n* ≥ 50	No. species with AUC & TSS > limit
Molluscs	609	106	99
Fish	473	243	220
Plants	209	196	178
Odonates	106	99	75
Crayfish	5	5	5
Sum	1,402	649	577

### 3.3 Thermal response curve types

Considering all categorizations of the air temperature variables, the most common TRC types for molluscs, fish, plants, odonates (Table 2) and crayfish (Supporting Information Tables S2.1 and S2.2) were Type I (unimodal symmetric response, 374 (Tmax_air_) and 437 (Tmean_air_) species) and Type IV (no response, 107 (Tmean_air_) and 179 (Tmax_air_) species) (Table 2). For example, for Tmean_air_, 58.6% (molluscs) to 89.3% (plants, odonates) had Type I as thermal response curve (Supporting Information Figure S1.2; Figure S1.3 for Tmax_air_; Table 2, Table S2.1 and S2.2). The Type IV response was mainly found for endemic and restricted‐range species. However, for *Marsilea batardae* endemic to the Iberian Peninsula we found a Type I response for the annual mean air temperature (Tmean_air_), suggesting that a Type IV response cannot be generalized for all endemic and restricted‐range species. A Type IV response consequently represents species with statistically no identifiable thermal preference.

**Table 2:** Thermal responses according to the univariate GAM using the annual mean air temperature and the maximum air temperature of the warmest month. *n* is the total number of species with the respective TRC and the corresponding percentage. Note that crayfish were excluded because of the low frequency of analyzed species

No.	Thermal response curve type		Taxonomic groups							
			Molluscs		Fish		Plants		Odonates	
			Tmean_air_	Tmax_air_	Tmean_air_	Tmax_air_	Tmean_air_	Tmax_air_	Tmean_air_	Tmax_air_
I	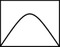	*n*	58	43	148	109	159	156	67	61
		%	58.6	43.4	67.3	49.5	89.3	87.6	89.3	81.3
II	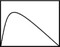	*n*	0	1	0	0	0	0	1	2
		%	0.0	1.0	0.0	0.0	0.0	0.0	1.3	2.7
III	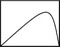	*n*	5	1	13	6	7	6	0	0
		%	5.1	1.0	5.9	2.7	3.9	3.4	0.0	0.0
IV	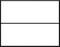	*n*	36	54	59	105	9	12	3	8
		%	36.4	54.5	26.8	47.7	5.1	6.7	4.0	10.7
V	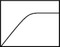	*n*	0	0	0	0	0	0	1	1
		%	0.0	0.0	0.0	0.0	0.0	0.0	1.3	1.3
VI	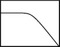	*n*	0	0	0	0	0	4	0	0
		%	0.0	0.0	0.0	0.0	0.0	2.2	0.0	0.0
VII	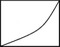	*n*	0	0	0	0	0	0	3	3
		%	0.0	0.0	0.0	0.0	0.0	0.0	4.0	4.0
VIII	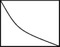	*n*	0	0	0	0	3	0	0	0
		%	0.0	0.0	0.0	0.0	1.7	0.0	0.0	0.0
∑			99		220		178		75	

Note

Tmean_air_, Annual mean air temperature; Tmax_air_, Maximum air temperature of the warmest month.

With regard to the species’ TRCs spatial distribution, the number of species with a unimodal thermal response curve type, that is, Type I‐III, decreased from central toward northern and southern Europe (Supporting Information Figure S1.4).

### 3.4 Assessment of species’ thermal properties

**Figure 2:**
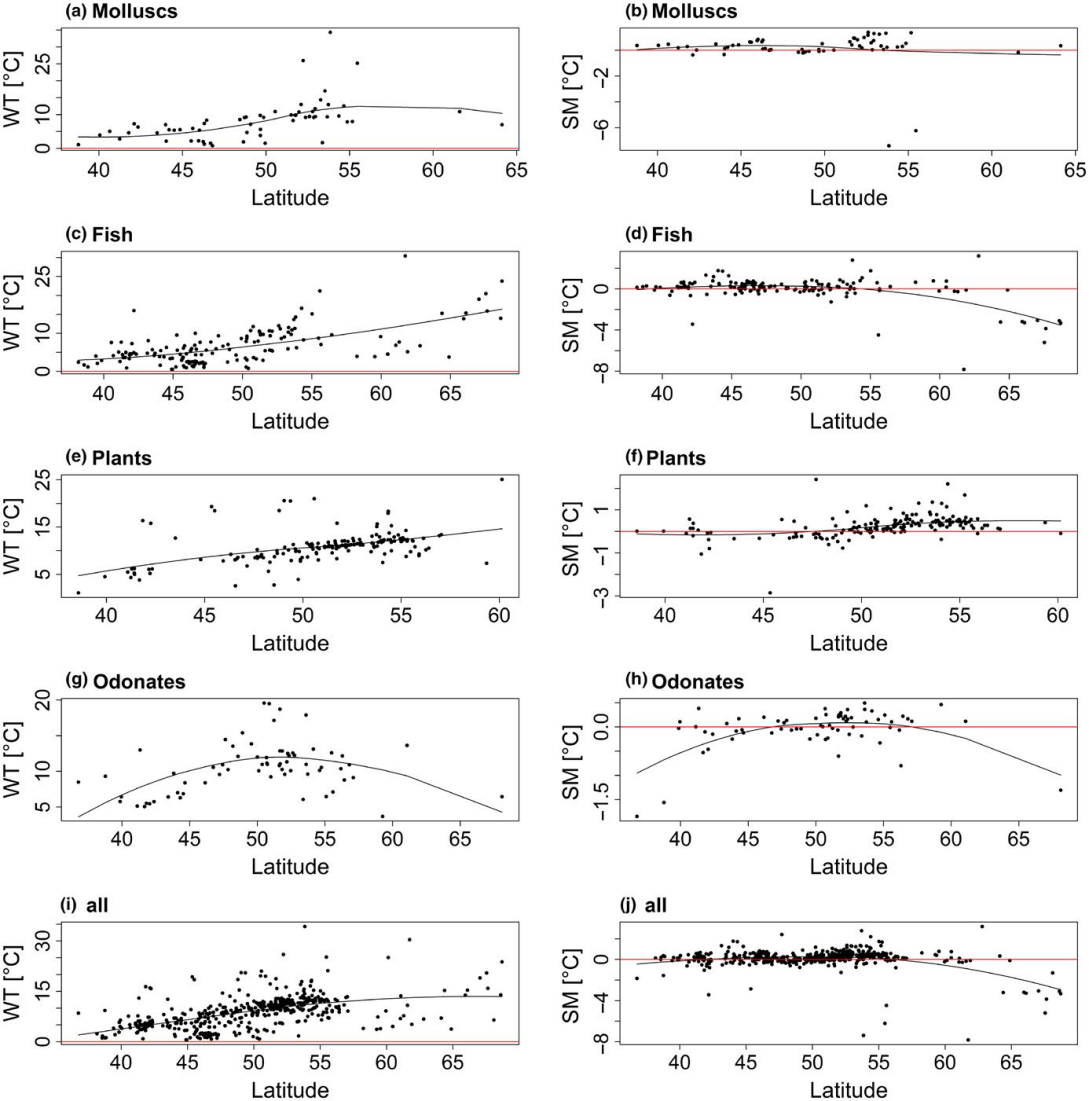
Latitudinal distributions and nonlinear trend lines of warming tolerance (WT = CT − *T*_pref_) and safety margin (SM = *T*_pref_− *T*_av_) for freshwater species inferred from the temperature variable Tmean_air_. CT represents the maximum temperature of a species’ occurrence, *T*_pref_ the temperature corresponding to the highest probability of occurrence and *T*_av_ the average temperature of the current distribution range. WT and SM were only computed for species with a unimodal response, that is, responses for which a temperature that maximizes the probability of occurrence could be determined. Here, latitude values correspond to the average latitude of each species’ European latitudinal range. WT, SM, and average latitude values were determined for (a, b) molluscs, (c, d) fishes, (e, f) plants, (g, h) odonates, and (i, j) all taxonomic groups with unimodal response curves combined. Note that crayfish were excluded because of the low frequency of analyzed species. Each dot represents the WT and SM of one species in the respective figure

**Figure 3:**
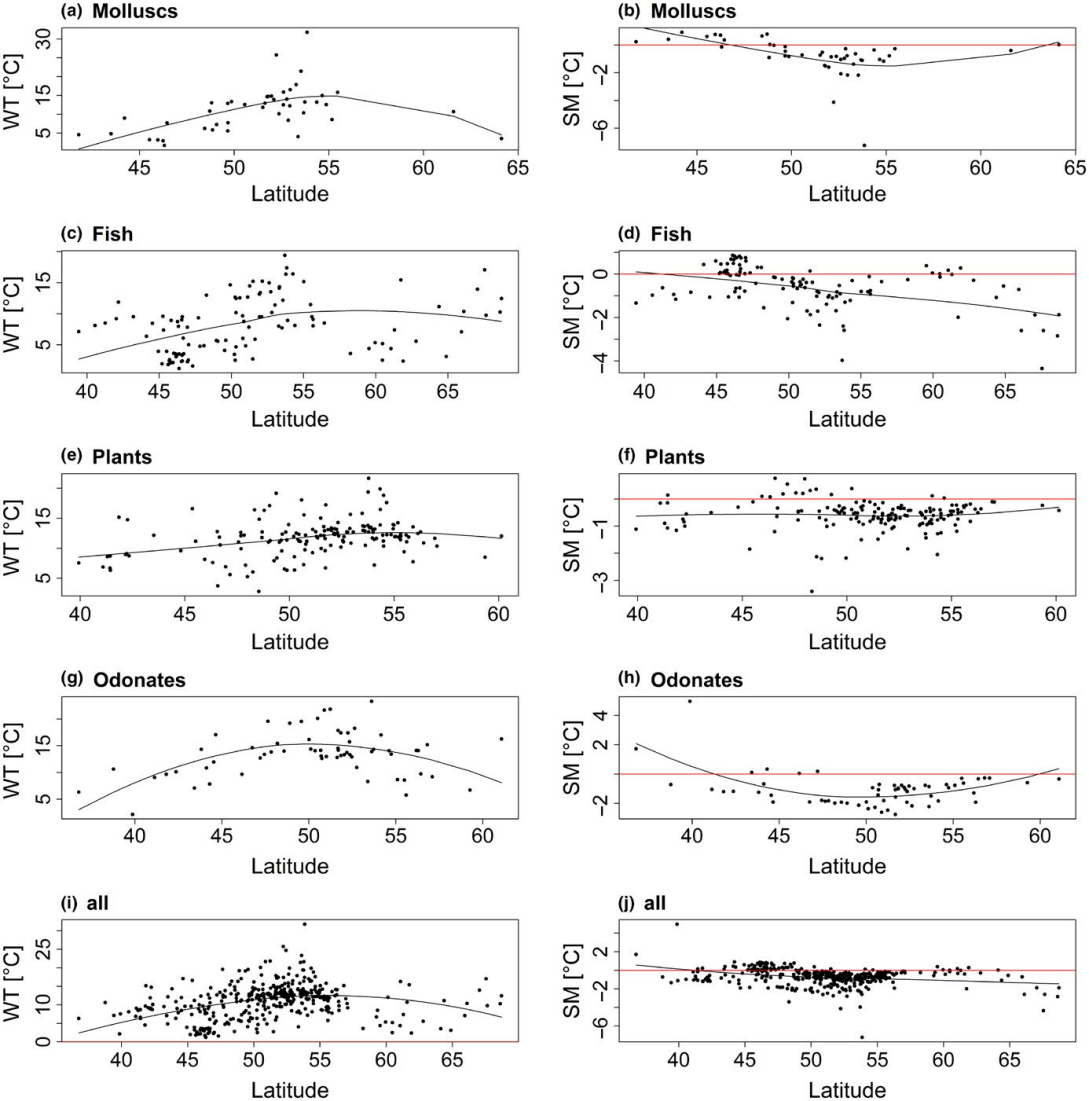
Latitudinal distributions and nonlinear trend lines of warming tolerance (WT = CT − *T*_pref_) and safety margin (SM = *T*_pref_− *T*_av_) for freshwater species inferred from the temperature variable Tmax_air_. CT represents the maximum temperature of a species’ occurrence, *T*_pref_ the temperature corresponding to the highest probability of occurrence and *T*_av_ the average temperature of the current distribution range. WT and SM were only computed for species with a unimodal response, that is, responses for which a temperature that maximizes the probability of occurrence could be determined. Here, latitude values correspond to the average latitude of each species’ European latitudinal range. WT, SM, and average latitude values were determined for (a, b) molluscs, (c, d) fishes, (e, f) plants, (g, h) odonates, and (i, j) all taxonomic groups with unimodal response curves combined. Note that crayfish were excluded because of the low frequency of analyzed species. Each dot represents the WT and SM of one species in the respective figure

Thermal responses were unimodal (Type I‐III) for 463 (80.2%) and 390 (67.6%) species using Tmean_air_ and Tmax_air_ to model species distributions, respectively (Table 2, Supporting Information Tables S2.1 and S2.2). For these species, the preferred temperature (T_pref_), warming tolerance (WT) and safety margin (SM) could be determined. Since high latitude analyses are based on a small number of species, high latitude WTs and SMs should be treated with caution. WT—latitude relationships were characterized by a WT increase with increasing latitude until around 55°N (Figures 2 and 3). As mentioned, for latitudes above 55°N, no reliable trend can be outlined because of the low number of species representing the higher latitudes. The SMs of all considered species were located around 0°C for 40°–55°N with species having either positive or negative SMs (Figures 2j and 3j). Both, WT and SM, were generally below 5°C for species with an average latitude of occurrence below 45°N for Tmean_air_ (e.g., the “Vulnerable” Iberian molluscs *Unio tumidiformis* with WT = 1.1°C, SM = 0.4°C (Supporting Information Tables S2.1 and S2.2)). Species living in regions >55°N had safety margins of down to around −7°C (e.g., the pea clam *Pisidium casertanum*with a safety margin of −7.4 and −7.2°C for Tmean_air_ and Tmax_air_, respectively) (Figures 2b and 3b; Supporting Information Table S2.1 and S2.2). Of those species with a unimodal response, the proportions of species with a negative safety margin per taxa group were between 22% (molluscs) and 44% (odonates) (Supporting Information Figure S1.5). We note that for Tmax_air_ the proportion of species with negative SMs was higher than for Tmean_air_, ranging from 70% (fish) to 91% (plants) (Supporting Information Figure S1.6).

**Figure 4:**
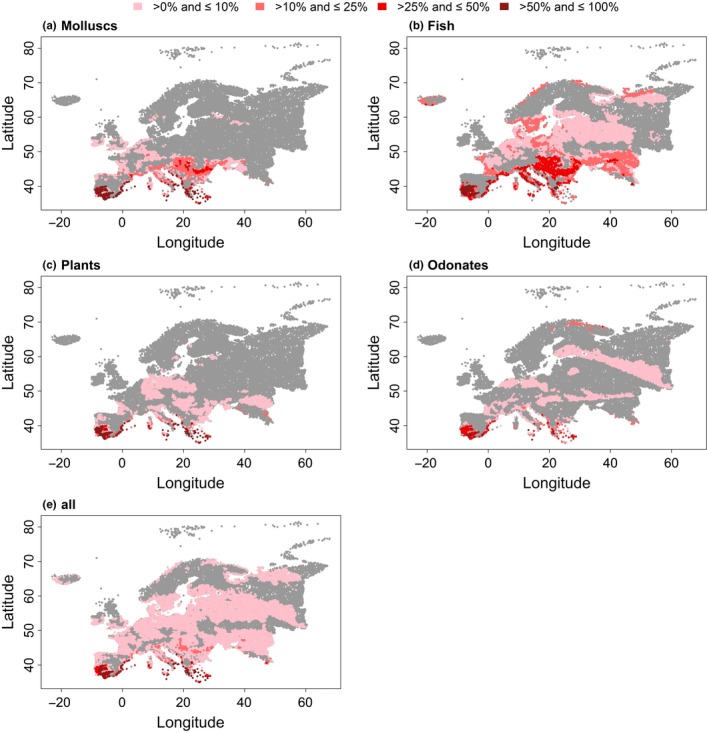
Relative frequency per catchment of species with the critical maximum temperature (CT) inferred from Tmean_air_ that is exceeded by the averaged projected temperature of the three climate models MOHC, IPSL, and MPI for the 2050 s for (a) molluscs, (b) fishes, (c) plants, (d) odonates, and (e) all taxonomic groups combined. The grey area represents either no occurrence or catchments in which the CT, that is, the maximum temperature of a species’ occurrence, is not exceeded by the projected temperatures. Note that crayfish were excluded because of the low frequency of analyzed species

**Figure 5:**
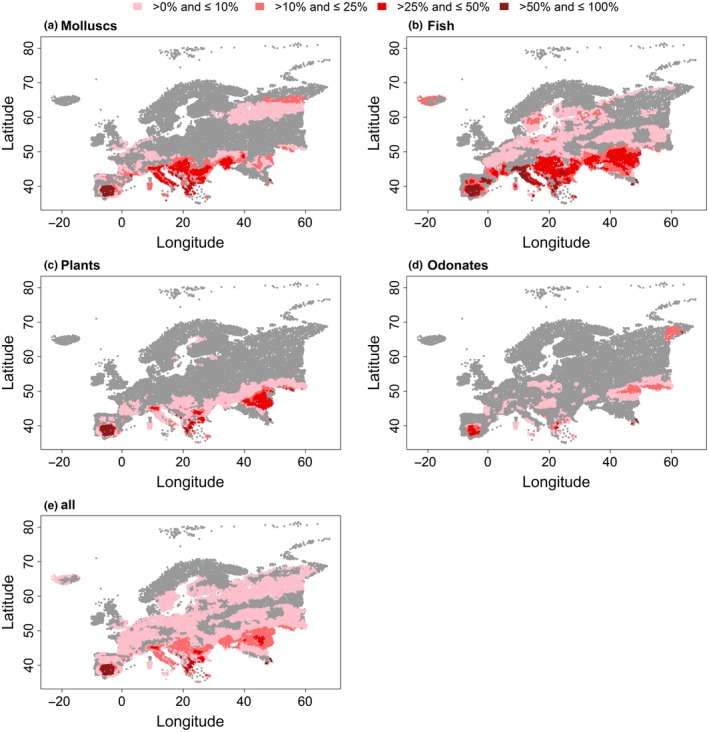
Relative frequency per catchment of species with the critical maximum temperature (CT) inferred from Tmax_air_ that is exceeded by the averaged projected temperature of the three climate models MOHC, IPSL, and MPI for the 2050 s for (a) molluscs, (b) fishes, (c) plants, (d) odonates, and (e) all taxonomic groups combined. The gray area represents either no occurrence or catchments in which the CT, that is, the maximum temperature of a species’ occurrence, is not exceeded by the projected temperatures. Note that crayfish were excluded because of the low frequency of analyzed species

For both air temperature variables, the analyses showed that areas in Spain and Mediterranean coastlines will be affected the most by rising temperatures (Figures 4e and 5e). Regarding the CT deduced from Tmax_air_, regions in Eastern Europe, especially in the coastal area of the Caspian Sea and the Danube region will likely suffer from temperature increases (Figure 5e). Among the studied taxonomic groups rising temperatures will be most detrimental to fish with more than 25% of the species in the respective catchments having a CT below the predicted temperatures mostly in the southern areas of Europe, reaching from the coastlines of Portugal and Spain to the coastlines of the Caspian Sea (Figures 4 and 5). Overall, the relative frequency of species with a critical difference between the projected and current maximum temperature of occurrence in a catchment was mainly below 10% (Figures 4e and 5e). These numbers were generally exceeded in the coastal areas of Spain and Italy, in south‐west Portugal, in coastal areas of Greece, in the Alpine region, the Balkans, and the western areas of the Caspian Sea.

## 4 DISCUSSION

Since decades, the classical Gaussian response curve, which has a single optimum and a decreasing probability of occurrence away from the optimum along the thermal gradient, is a well‐accepted assumption for a species’ thermal response (Gauch & Whittaker, 1972). Thermal response curves of the European freshwater species did not vary greatly among taxonomic groups and the species within a group. Our results highlighted that the unimodal response curves (Type I–III) were most frequent among all considered taxonomic groups and all four temperature variables (Tmean_air_—annual mean air temperature, Tmax_air_—maximum air temperature of the warmest month, Tmean_water_—annual mean water temperature, Tmax_water_—maximum water temperature of the warmest month) that were used to model the thermal response (390–463 of 577 species, i.e., 67.6%–80.2%). High pairwise correlations of the temperature variables (above 0.8) explain the similarities of the results. For species with spatial distribution ranges characterized by substantially differing thermal gradients across the used temperature metrics, the corresponding thermal response types (TRCs) also varied. Species with unimodal response types were most common in central Europe, following thus the species richness patterns. Namely the species density was higher in central than in northern and southern Europe.

Despite the “overarching importance of thermal regimes to aquatic life” (Isaak et al., 2017), thermal niches of freshwater species are only scarcely studied. For example, Lassalle, Béguer, Beaulaton, and Rochard (2008) found unimodal responses using annual temperature for *Acipenser gueldenstaedtii*, *Acipenser stellatus*, *Alosa alosa*, *Alosa tanaica*, *Vimba vimba*, and *Osmerus eperlanus*, corresponding to our response type categorization for Tmean_air_. The response type of the cold‐water specialist brown trout (*Salmo trutta*, Type III for Tmax_water_) coincides with the findings of Isaak et al. (2017), where both the multivariate and univariate (using August stream temperature as explanatory variable) models showed a unimodal response. For the fish species investigated by Logez et al. (2012) using the mean air temperature in July, response curves for Tmax_air_ are different for three fish species (*Alburnus alburnus*, *Rhodeus amarus*, *Salmo trutta*). Differences may have resulted from consideration of native portions of species ranges by Logez et al. (2012), whereas our study considers global species ranges.

Warming tolerances and safety margins for the different temperature variables showed only marginal differences in the latitudinal trends. The species‐based warming tolerance increased by moving northwards until 55°N, indicating that on average species in central Europe had a greater difference between the critical and preferred temperature than southern species and thus a greater capacity to cope with warming. For high latitude species, no reliable latitudinal relationships above approximately 55°N could be given due to a low species number as compared to much larger data availability for southern to central Europe. In addition, due to the statistical barrier of 50 occurrences at the analyzed scale, many endemic species of the Italian, Iberian, and Balkan Peninsulas with few catchment occurrences could not be included for southern parts of Europe. Most species in central Europe have a high colonization capability, wider distribution ranges and experience greater intra‐annual variability than species at lower latitudes, which explains their warming tolerance. Furthermore, while some specialists of cold climates in the far north had also a low warming tolerance comparable to species in the south (e.g., *Gasterosteus islandicus* native to Iceland with WT = 3.8°C and WT = 3.1°C inferred from Tmean_air_ and Tmax_air_, respectively), other species with average latitudes located in colder climates (>60°N) had higher warming tolerances. High warming tolerance at these latitudes may be an indication of species occurrences in areas different from the European ranges. For example, the fish species *Osmerus dentex* (WT = 19°C and WT = 14°C inferred from Tmean_air_and Tmax_air_, respectively) occurs at the European scale at an average latitude of 67°N, but is also found at lower latitudes, for example, in Japan or Korea. Despite the fact that WT increased with latitude up to about 55°N, one has to be cautious with the interpretation of this result as the preferred temperature, *T*_pref_, might have been already exceeded for some species (negative safety margin).

Shuter and Post (1990) and Brazner et al. (2005) found that temperature is one of the main drivers of the spatial distribution of stream fish, suggesting high vulnerability to future temperature rise. In our study, future temperature predictions showed that especially fish will be affected critically by rising temperatures. Fishes spend their entire life cycle in the water. Consequently, they depend on the water temperature throughout all life stages, in contrast to merolimnic species (e.g., odonates) that are connected to the waters only in early stages of their life cycle, having the ability to escape water temperature rises in critical periods. Additionally, sensitivity of fishes to temperature changes (Magnuson, Crowder, & Medvick, 1979), in terms of survival and growth, underline the threat fishes are facing in the future. Potential movement is connected with a maximization of the growth rate (Jobling, 1981) and metabolic power available for reproduction and activity (Kelsch, 1996) and may vary by life‐history strategies, for example, migratory and sedentary fish. Considering all taxonomic groups, especially the Balkans, the western area of the Caspian Sea and the coastal areas of the Mediterranean Sea like southern Portugal and Spain or Italy and Greece will be affected according to the temperature projections. Additional changes in the marine realm (Lejeusne, Chevaldonne, Pergent‐Martini, Boudouresque, & Perez, 2010) demonstrate the ongoing and upcoming changes in the Mediterranean area. More than 25% of the considered species in the catchments of these regions had a CT below the predicted temperature of the 2050s. Some species can adapt, more or less fast, to a certain extent by physiological adjustment (Johnson & Kelsch, 1998) or behavioral thermoregulation (Heggenes, Krog, Lindås, Dokk, & Bremnes, 1993), while another option for escaping or mitigating these threatening conditions is movement to suitable areas. However, especially in the regions of the Iberian Peninsula and the Mediterranean area, where thermal alteration impacts will be the strongest, endemic, or restricted‐range species prevail. The latter suggest an urgent need for further research on species’ sensitivity to climate warming; in particular, effects of rising temperatures have to be investigated in the context of species thermal properties, with the focus on species with currently small thermal ranges, and dispersal traits paired with habitat suitability and connectivity.

Strengths and weaknesses of statistical models describing species distributions have been extensively evaluated in the literature (see, e.g., Franklin, 2009). Considering our study, the thermal response curves and thus the occurrence probabilities along thermal gradients resulting from GAMs should be viewed in the context of the analyzed scale (catchments) and statistical approach. Consequently, different thermal responses may result from local scale data and for species with few occurrences (*n* < 50) thermal responses could not be captured. Thus, high‐endemism areas (peri‐Mediterranean region and Balkans) are in need of additional extensive analyses at finer scales. Furthermore, our thermal response curves do not consider the above discussed possibility of adaption to environmental changes. We considered annual mean water temperature and the maximum water temperature of the warmest month as a transformation of the corresponding air temperature via a global relationships model (Punzet et al., 2012). The key shortcoming of the latter model is that it solely depends on air temperature and thus ignores effects such as catchment heterogeneity, shading, or dissolved oxygen concentration. Although thermal responses give a quantification of thermal habitats (Hester & Doyle, 2011) and a necessary assessment of the impact of future global warming (Vetaas, 2000), they do not account for other environmental and community influences. It is important to keep in mind that species do not respond to a single environmental factor (Økland, 1992). Therefore, our results on thermal properties and responses should be viewed in the context of complex interactions of different factors. For example, Verberk, Durance, Vaughan, and Ormerod (2016) outlined effects of stream oxygenation on thermal tolerances, while Arismendi, Safeeq, Johnson, Dunham, and Haggerty (2012) found that the combination of flow reduction and temperature increase could lead to an exacerbation of the reduction in cold‐water species habitat. The latter leads to a process known as “thermophilization,” describing the increasing dominance of warm‐water species (De Frenne et al., 2013). As such, amplifications of climate change related impacts caused by anthropogenic pressures, for example, intensified eutrophication of lake catchments and especially the disappearance of water bodies and modification of habitats (Nowakowski, Thompson, Donnelly, & Todd, 2017) should be further considered in the context of the potential future species distributions. New generations of species distribution models aim at combining abiotic and biotic factors, but need detailed and thus rarely available ecological information about species for reliable projections (Singer et al., 2016; Urban et al., 2016).

In summary, future temperatures are expected to exceed the current maximum temperature of occurrence of species living in coastal areas of the Mediterranean Sea, the Balkans, and the western area of the Caspian Sea. Synergetic effects of rising temperatures and other influencing factors, such as restricted catchment connectivity or anthropogenic disturbances in these areas, will additionally aggravate the viability of populations, but the whole scope of climate change impacts remains difficult to grasp. However, given the high vulnerability of freshwater ecosystems to climate change, re‐assessments of the existing conservation areas and integrated management practices that facilitate species migration are urgently needed. Furthermore, for keeping the thermal habitat suitability of European catchments within species tolerance limits, a renewed effort to slow down the pace of climate change is essential.

## AUTHOR'S CONTRIBUTION

OK and DM designed the study and collected the data. OK ran the models and prepared the figures and tables with input from DH, KF, and DM. OK and DM wrote the first draft of the manuscript. All authors contributed to revisions.

## DATA ACCESSIBILITY

Data on European freshwater species were collected during the EU‐funded research project BioFresh and are available at https://www.iucnredlist.org/technical-documents/spatial-data. More information is available at https://project.freshwaterbiodiversity.eu/.

Gridded climate data for the second half of the 20th century were extracted from the WorldClim data set and are available at www.worldclim.org.

Data on future climate projections were gathered from the CIAT (International Center for Tropical Agriculture) dataset available at www.ccafs-climate.org.
