# Supplementary material for: Freshwater species distributions along thermal gradients
Source: Ecol Evol. 2018 Dec 18;9(1):111–24. doi: 10.1002/ece3.4659 (PMC6342105; doi:10.1002/ece3.4659)
Supplement: Supplementary file 1 [file ECE3-9-111-s001.docx]

**Appendix S1**

Freshwater Species Distributions along Thermal Gradients

Oskar Kärcher, Daniel Hering, Karin Frank, Danijela Markovic

**Table S1.1** Pairwise Pearson correlation coefficients among the used temperature variables (Tmean_air_ – Annual mean air temperature; Tmax_air_ – Maximum air temperature of the warmest month; Tmean_water_ – Annual mean water temperature; Tmax_water_ – Maximum water temperature of the warmest month).

| **Variable** | Tmean_air_ | Tmax_air_ | Tmean_water_ | Tmax_water_ |
| --- | --- | --- | --- | --- |
| Tmean_air_ | 1.00 | 0.88 | 0.98 | 0.86 |
| Tmax_air_ |  | 1.00 | 0.86 | 0.96 |
| Tmean_water_ |  |  | 1.00 | 0.81 |
| Tmax_water_ |  |  |  | 1.00 |

**Table S1.2** Thermal responses according to the univariate GAM using the annual mean water temperature and the maximum water temperature of the warmest month. $n$ is the total number of species with the respective TRC and the corresponding percentage. Note that crayfish were excluded because of the low frequency of analysed species.

| **No.** | **Thermal Response Curve**  **Type** | | **Taxonomic groups** | | | | | | | | |
| --- | --- | --- | --- | --- | --- | --- | --- | --- | --- | --- | --- |
|  |  |  | **Molluscs** | | **Fish** | | **Plants** | | | **Odonates** | |
|  |  |  | **Tmean**  **water** | **Tmax**  **water** | **Tmean**  **water** | **Tmax**  **water** | **Tmean**  **water** | **Tmax**  **water** | **Tmean**  **water** | | **Tmax**  **water** |
| I | **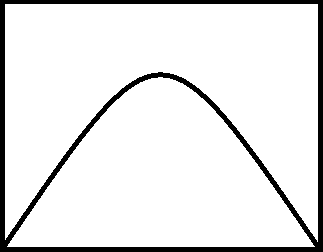** | $n$ | 53 | 39 | 149 | 109 | 162 | 137 | 67 | | 60 |
|  |  | $\boldsymbol{\%}$ | 53.5 | 39.4 | 67.7 | 49.5 | 91.0 | 77.0 | 89.3 | | 80.0 |
| II | **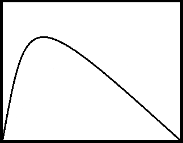** | $n$ | 0 | 0 | 0 | 0 | 0 | 0 | 1 | | 1 |
|  |  | $\boldsymbol{\%}$ | 0.0 | 0.0 | 0.0 | 0.0 | 0.0 | 0.0 | 1.3 | | 1.3 |
| III | **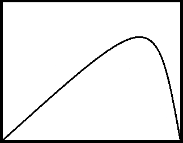** | $n$ | 3 | 6 | 11 | 8 | 3 | 25 | 0 | | 2 |
|  |  | $\boldsymbol{\%}$ | 3.0 | 6.1 | 5.0 | 3.6 | 1.7 | 14.0 | 0.0 | | 2.7 |
| IV | **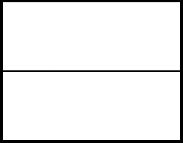** | $n$ | 43 | 54 | 60 | 103 | 10 | 12 | 3 | | 6 |
|  |  | $\boldsymbol{\%}$ | 43.4 | 54.5 | 27.3 | 46.8 | 5.6 | 6.7 | 4.0 | | 8.0 |
| V | **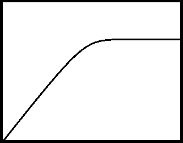** | $n$ | 0 | 0 | 0 | 0 | 0 | 0 | 2 | | 1 |
|  |  | $\boldsymbol{\%}$ | 0.0 | 0.0 | 0.0 | 0.0 | 0.0 | 0.0 | 2.7 | | 1.3 |
| VI | **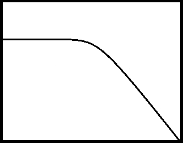** | $n$ | 0 | 0 | 0 | 0 | 0 | 4 | 0 | | 0 |
|  |  | $\boldsymbol{\%}$ | 0.0 | 0.0 | 0.0 | 0.0 | 0.0 | 2.2 | 0.0 | | 0.0 |
| VII | **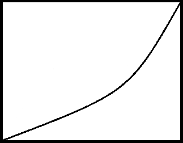** | $n$ | 0 | 0 | 0 | 0 | 0 | 0 | 2 | | 5 |
|  |  | $\boldsymbol{\%}$ | 0.0 | 0.0 | 0.0 | 0.0 | 0.0 | 0.0 | 2.7 | | 6.7 |
| VIII | **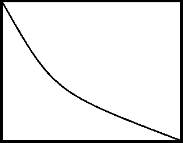** | $n$ | 0 | 0 | 0 | 0 | 3 | 0 | 0 | | 0 |
|  |  | $\boldsymbol{\%}$ | 0.0 | 0.0 | 0.0 | 0.0 | 1.7 | 0.0 | 0.0 | | 0.0 |
| $\sum$ |  | *n* | 99 | | 220 | | 178 | | | 75 | |

Tmean_water_ – Annual mean water temperature; Tmax_water_ – Maximum water temperature of the warmest month


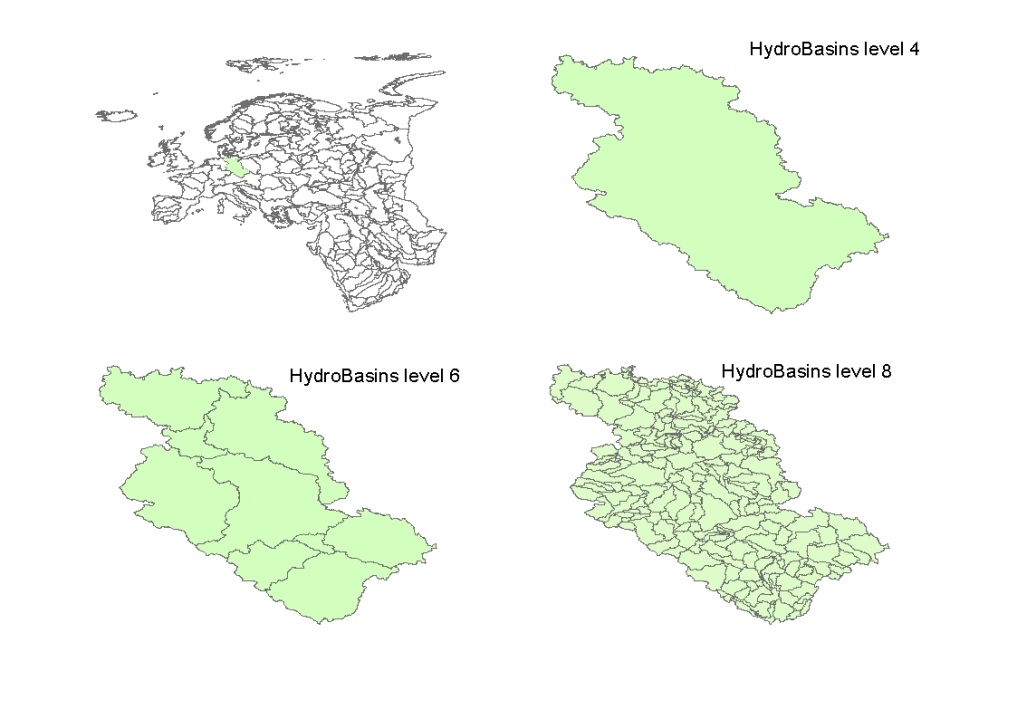


Fig. S1.1 Different HydroBasins dataset resolutions for the Elbe River Basin (green).

Fig. S1.2 Relative frequency of the different curve types for molluscs, fish, plants and odonates for Tmean_air_. Note that crayfish were excluded because of the low frequency of analysed species.

Fig. S1.3 Relative frequency of the different curve types for molluscs, fish, plants and odonates for Tmax_air_. Note that crayfish were excluded because of the low frequency of analysed species.


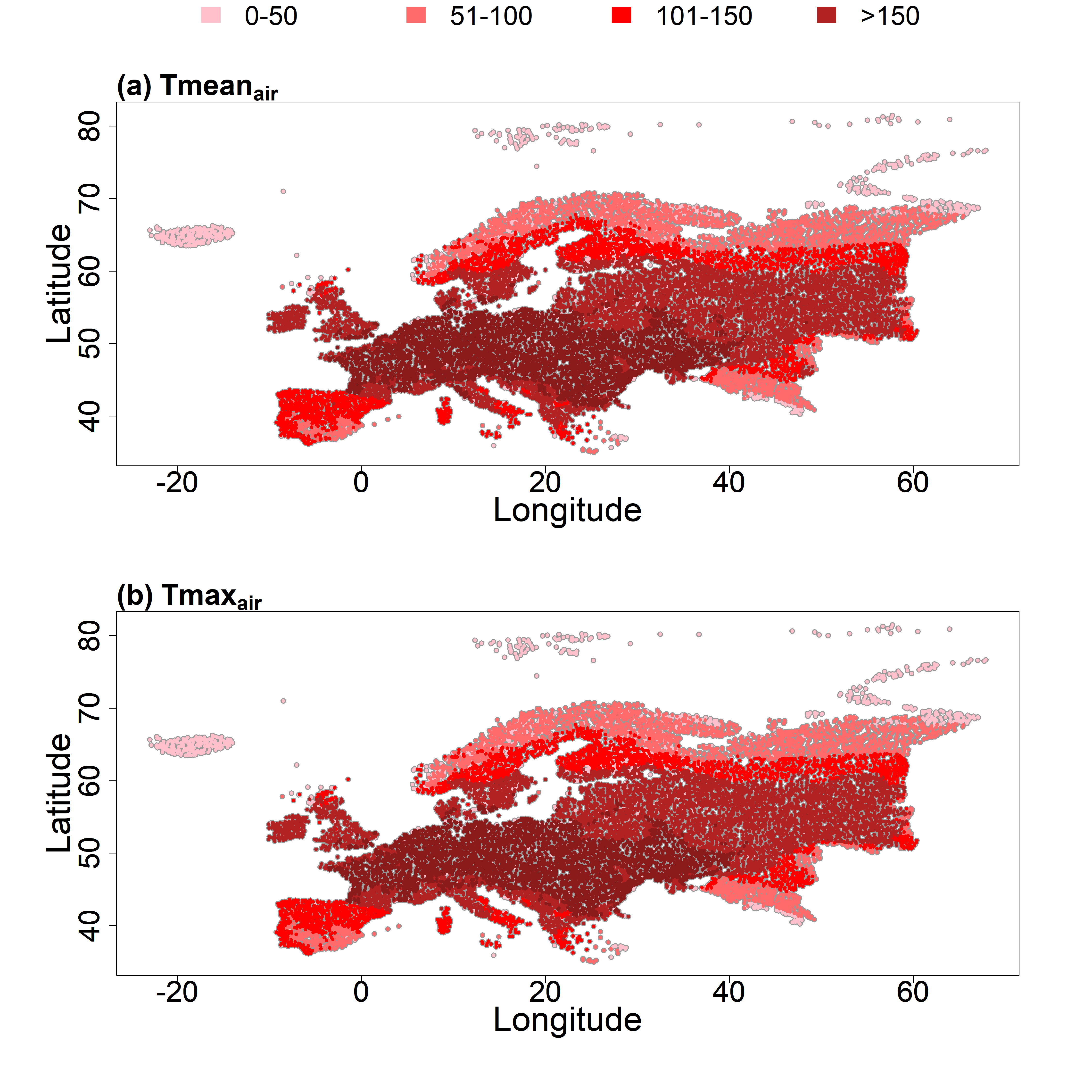


**Fig. S1.4** Variation in the number of species with a unimodal response per catchment for (a) Tmean_air_ and (b) Tmax_air_.


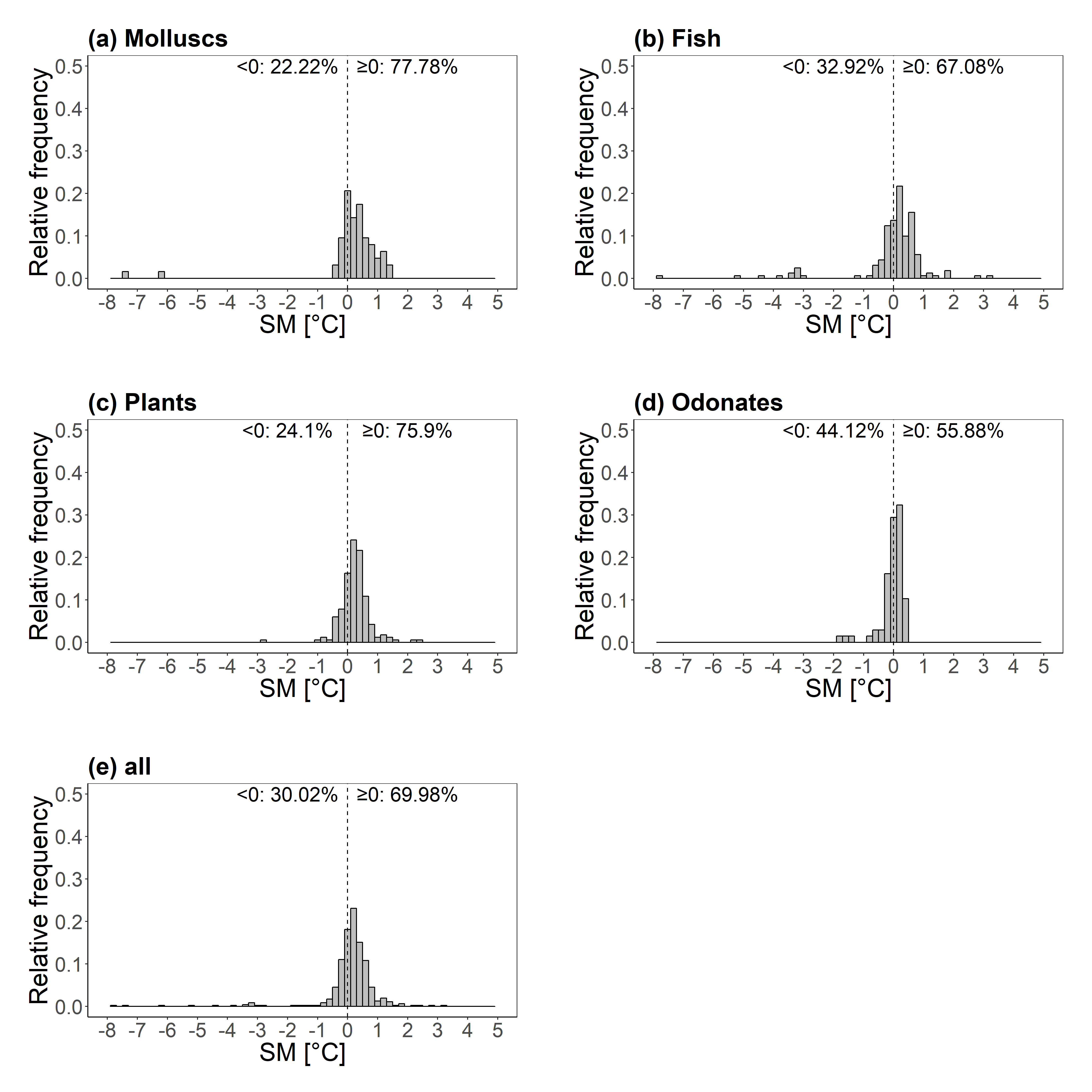


**Fig. S1.5** Relative frequencies of the safety margin (SM = T_pref_ – T_av_) distribution of unimodal species for (a) molluscs, (b) fishes, (c) plants, (d) odonates, and (e) all taxonomic groups combined inferred from Tmean_air_. The dotted line at 0°C separates negative and positive SMs with the relative frequency of the species of the corresponding taxonomic group having a negative or positive SM at the upper end. Note that crayfish were excluded because of the low frequency of analysed species.


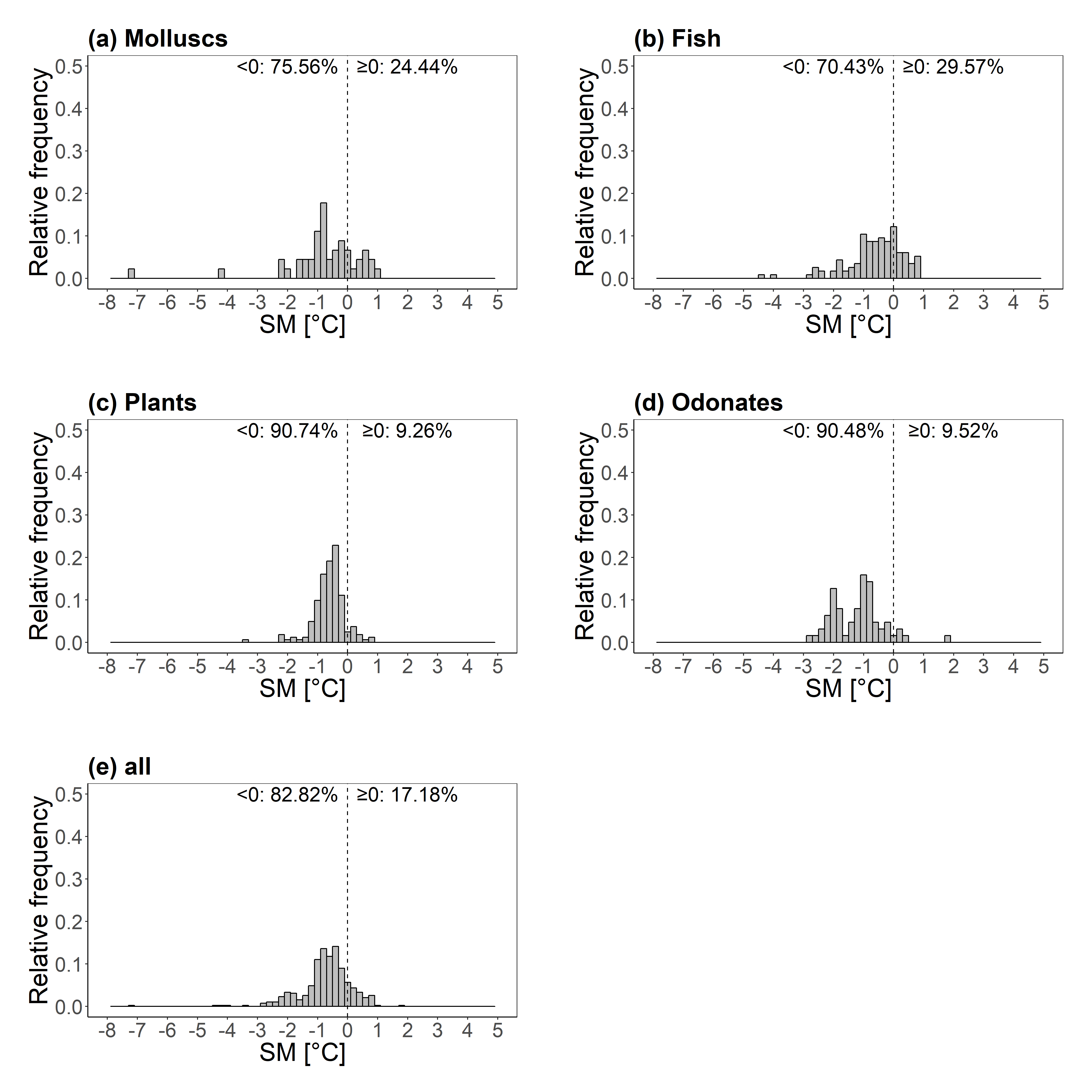


**Fig. S1.6** Relative frequencies of the safety margin (SM = T_pref_ – T_av_) distribution of unimodal species for (a) molluscs, (b) fishes, (c) plants, (d) odonates, and (e) all taxonomic groups combined inferred from Tmax_air_. The dotted line at 0°C separates negative and positive SMs with the relative frequency of the species of the corresponding taxonomic group having a negative or positive SM at the upper end. Note that crayfish were excluded because of the low frequency of analysed species.


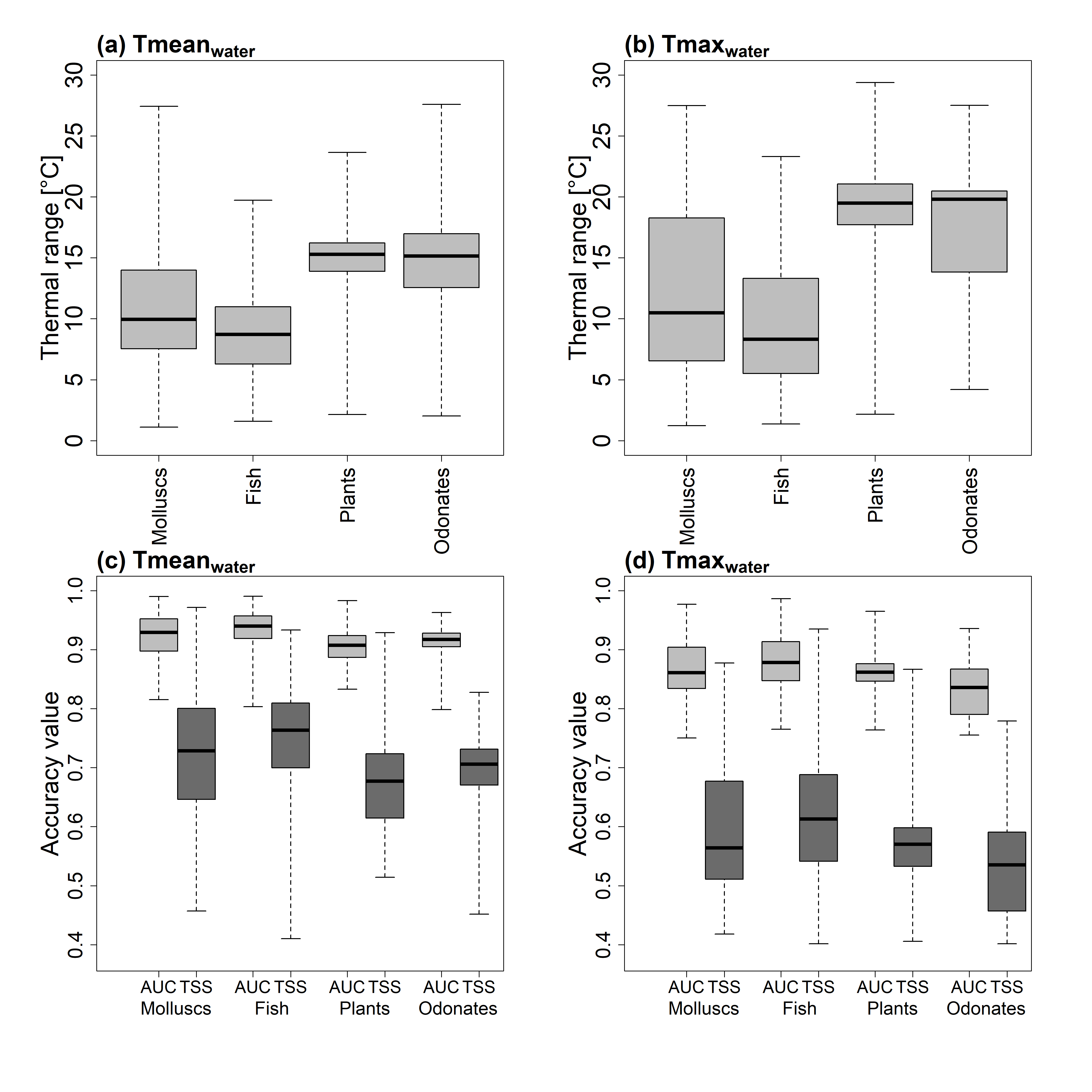
 **Fig. S1.7** Thermal ranges of the species and the distribution of the accuracy measures per taxonomic group for the respective temperature variable, i.e. for (a, c) Tmean_water_ and (b, d) Tmax_water_. The boxplots illustrate the distribution of the minimum, 25% quantile, median, 75% quantile and maximum of the thermal ranges. The minimum and maximum are displayed by the end of the corresponding whiskers. Note that crayfish were excluded because of the low frequency of analysed species.

**
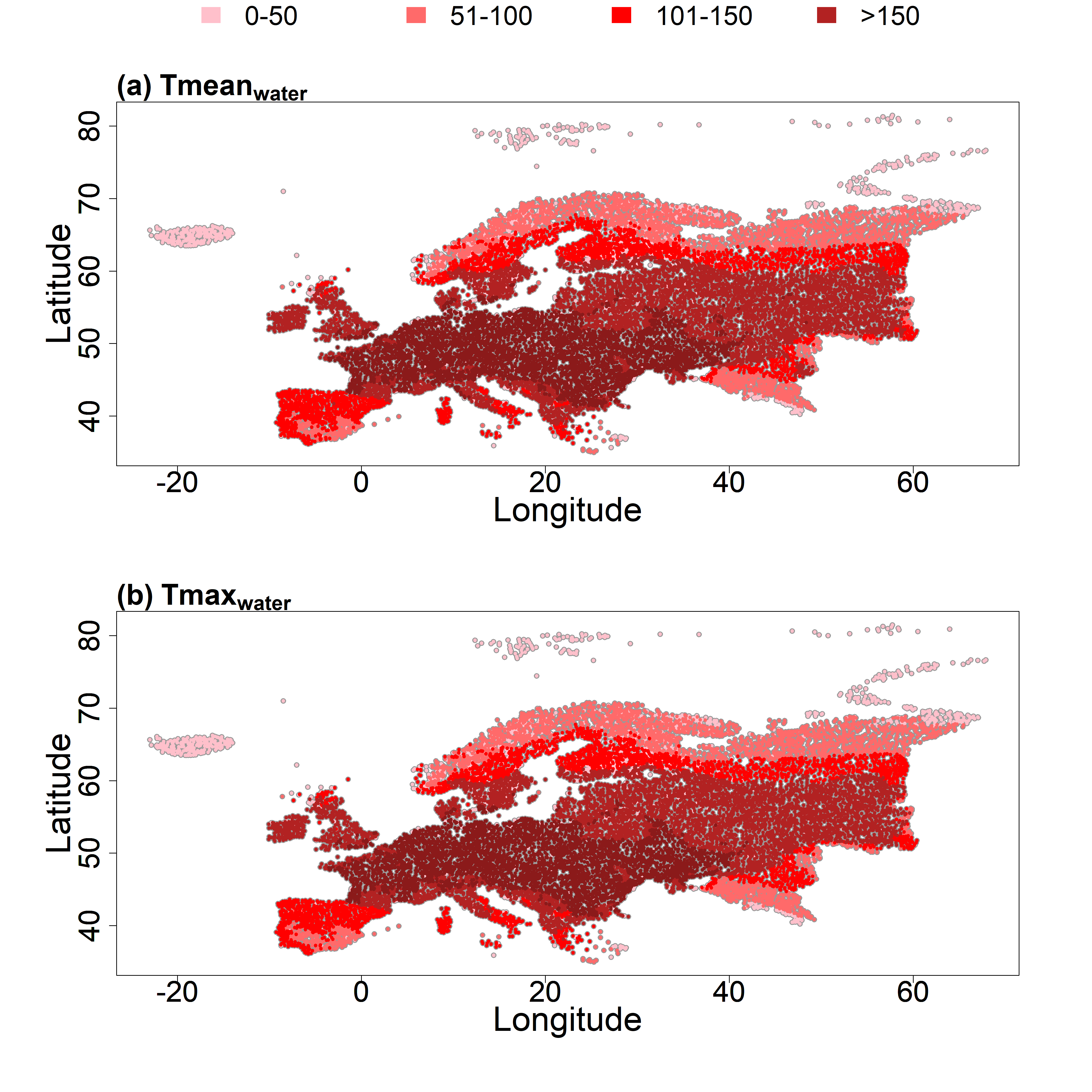
**

**Fig. S1.8** Variation in the number of species with a unimodal response per catchment for (a) Tmean_water_ and (b) Tmax_water_.

Fig. S1.9 Relative frequency of the different curve types for molluscs, fish, plants and odonates for Tmean_water_. Note that crayfish were excluded because of the low frequency of analysed species.

Fig. S1.10 Relative frequency of the different curve types for molluscs, fish, plants and odonates for Tmax_water_. Note that crayfish were excluded because of the low frequency of analysed species.


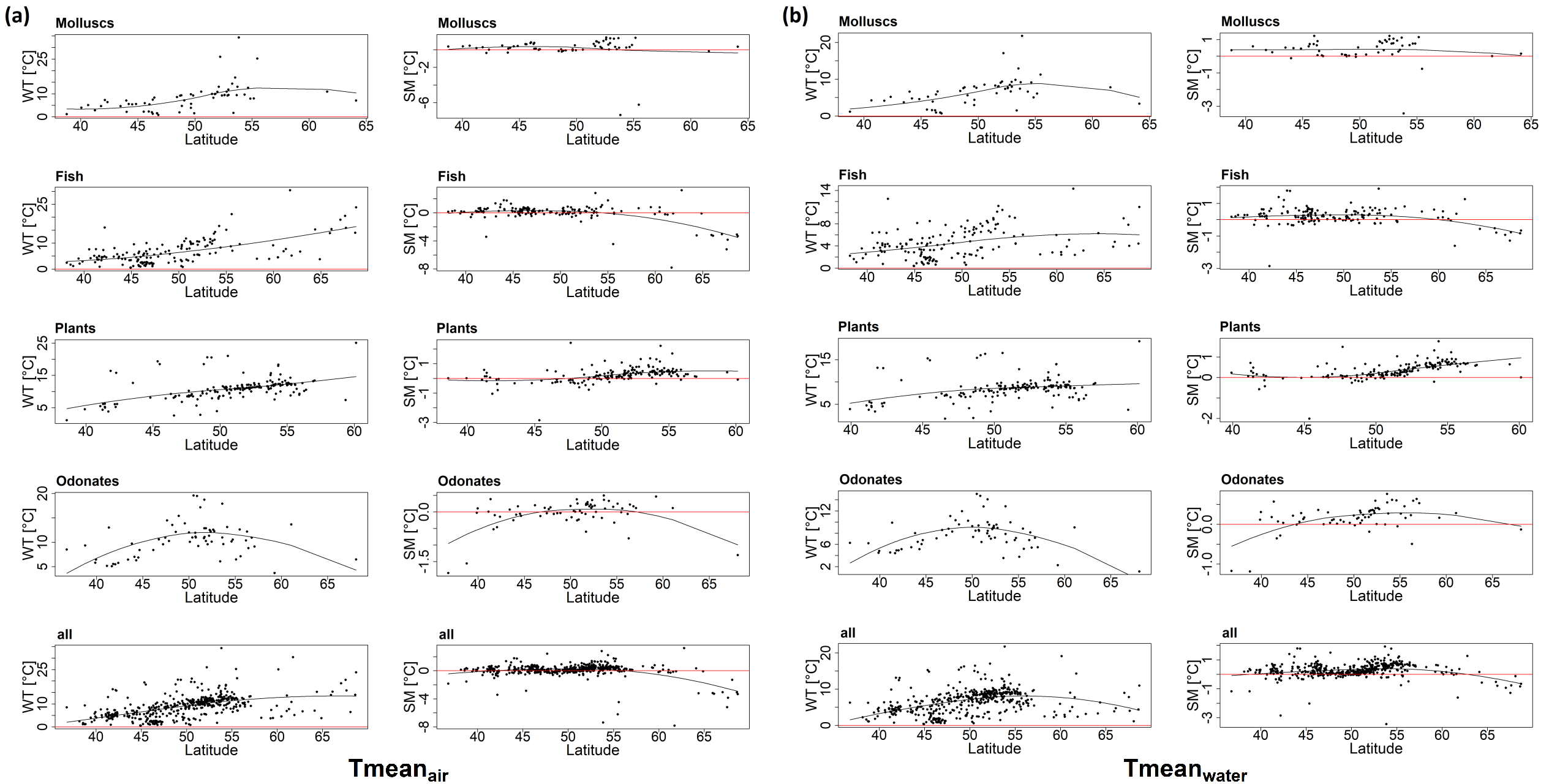


Fig. S1.11 Comparison of the latitudinal distributions and non-linear trend lines of the warming tolerance (WT = CT – T_pref_) and safety margin (SM = T_pref_ – T_av_) for freshwater species inferred from the temperature variables (a) Tmean_air_ and (b) Tmean_water_. WT and SM were only computed for species with a unimodal response. Here, latitude values correspond to the average latitude of each species’ European latitudinal range. Note that crayfish were excluded because of the low frequency of analysed species. Each dot represents the WT and SM of one species in the respective figure.


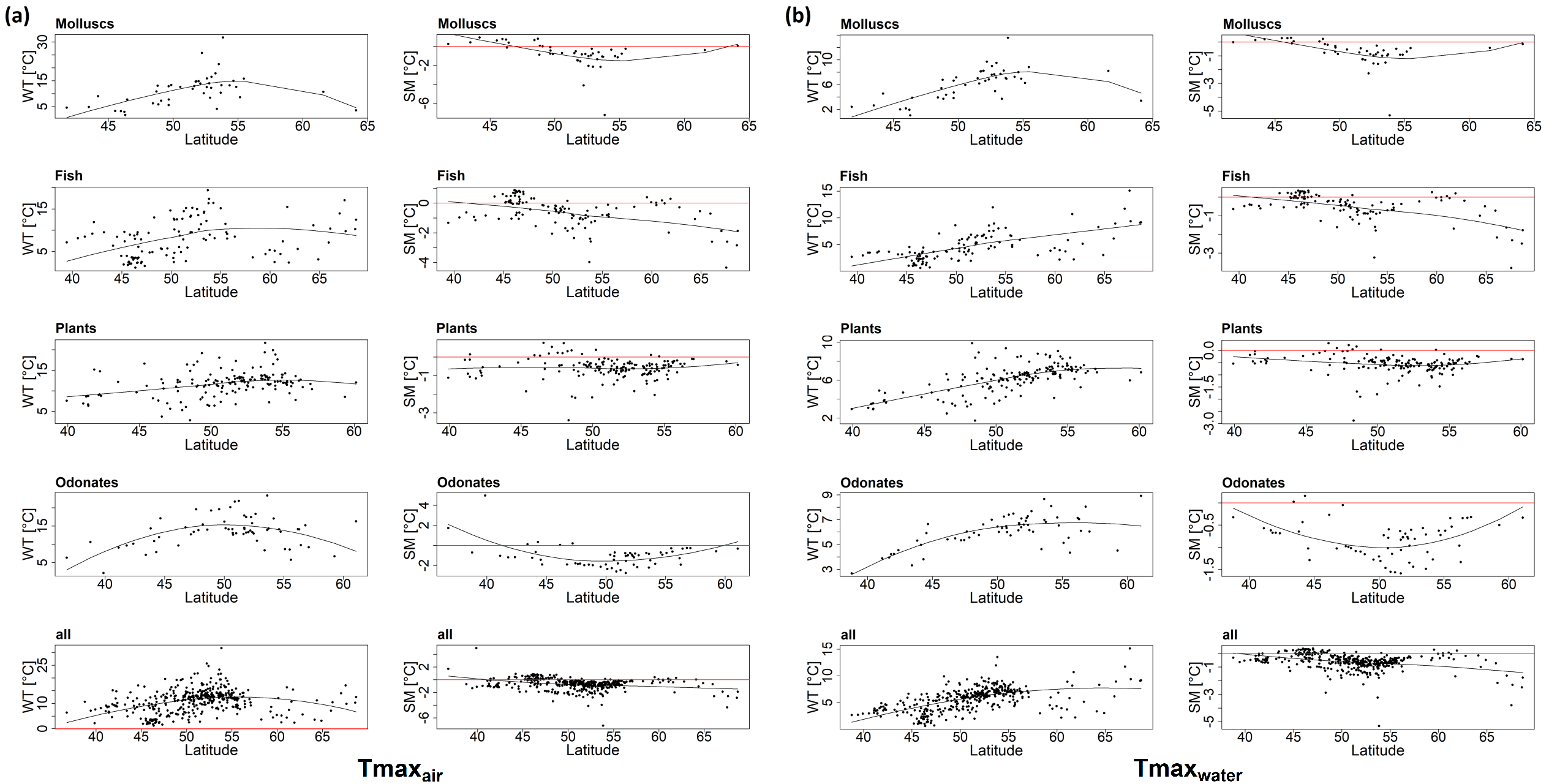


Fig. S1.12 Comparison of the latitudinal distributions and non-linear trend lines of the warming tolerance (WT = CT – T_pref_) and safety margin (SM = T_pref_ – T_av_) for freshwater species inferred from the temperature variables (a) Tmax_air_ and (b) Tmax_water_. WT and SM were only computed for species with a unimodal response. Here, latitude values correspond to the average latitude of each species’ European latitudinal range. Note that crayfish were excluded because of the low frequency of analysed species. Each dot represents the WT and SM of one species in the respective figure.


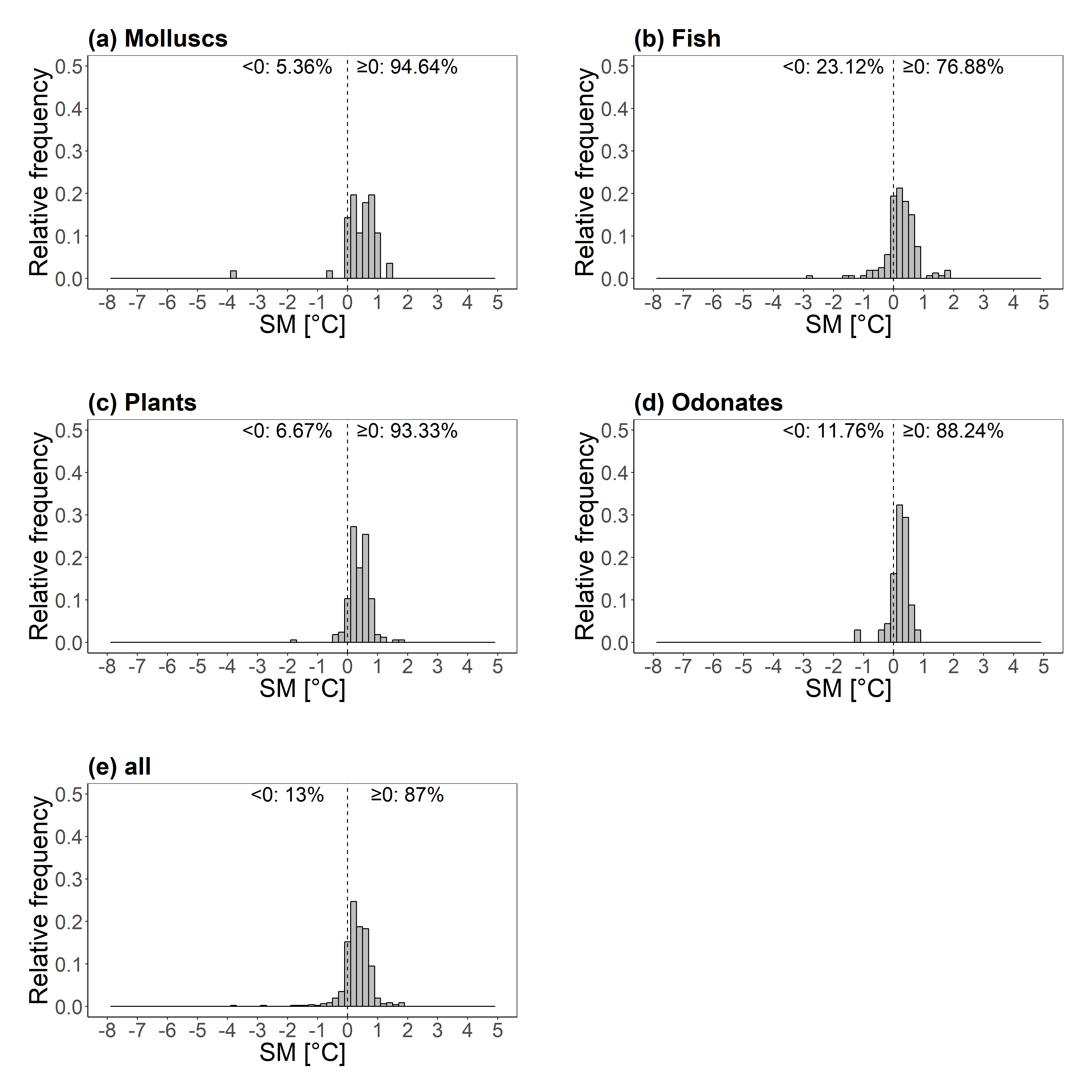


**Fig. S1.13** Relative frequencies of the safety margin (SM = T_pref_ – T_av_) distribution of unimodal species for (a) molluscs, (b) fishes, (c) plants, (d) odonates, and (e) all taxonomic groups combined inferred from Tmean_water_. The dotted line at 0°C separates negative and positive SMs with the relative frequency of the species of the corresponding taxonomic group having a negative or positive SM at the upper end. Note that crayfish were excluded because of the low frequency of analysed species.


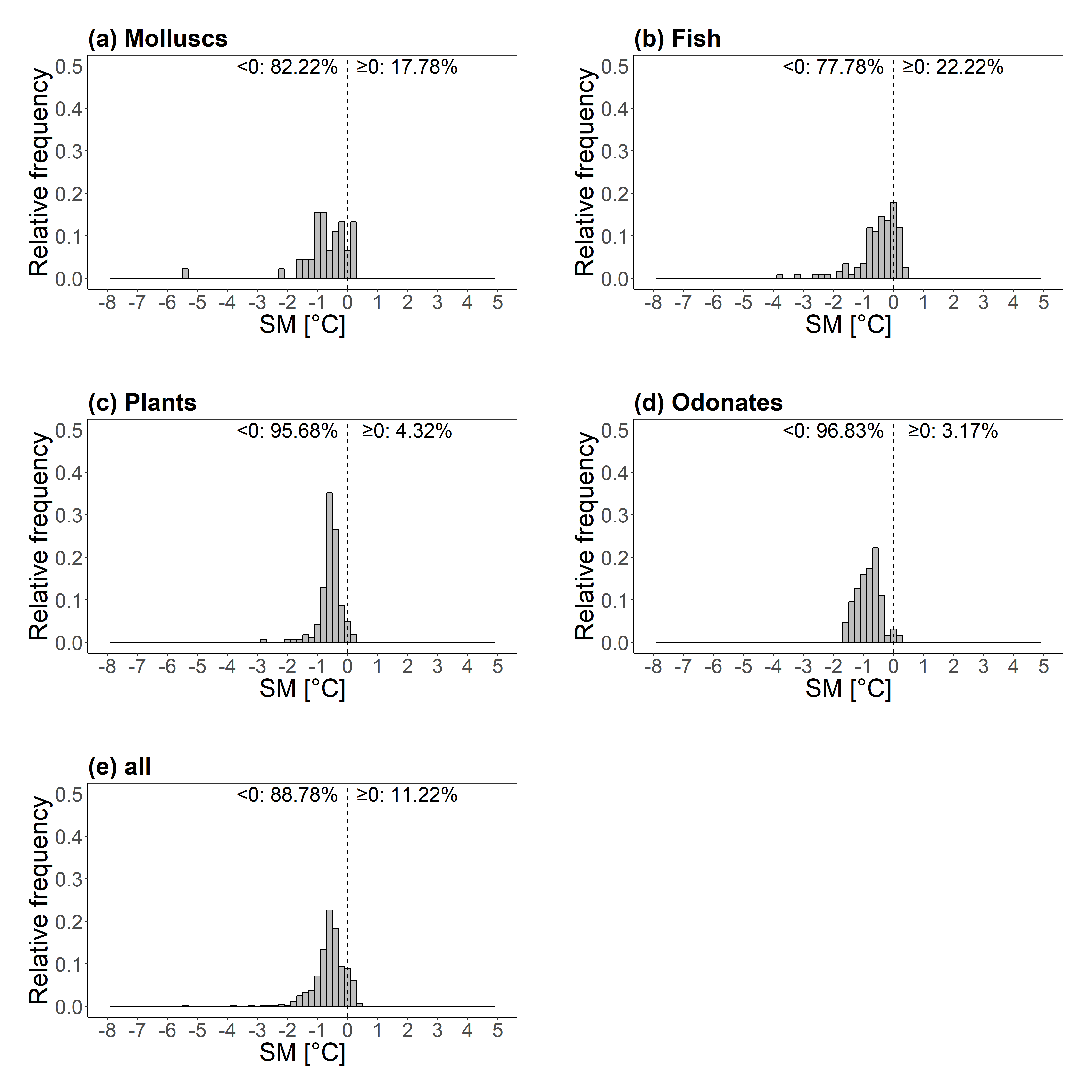


**Fig. S1.14** Relative frequencies of the safety margin (SM = T_pref_ – T_av_) distribution of unimodal species for (a) molluscs, (b) fishes, (c) plants, (d) odonates, and (e) all taxonomic groups combined inferred from Tmax_water_. The dotted line at 0°C separates negative and positive SMs with the relative frequency of the species of the corresponding taxonomic group having a negative or positive SM at the upper end. Note that crayfish were excluded because of the low frequency of analysed species.

**
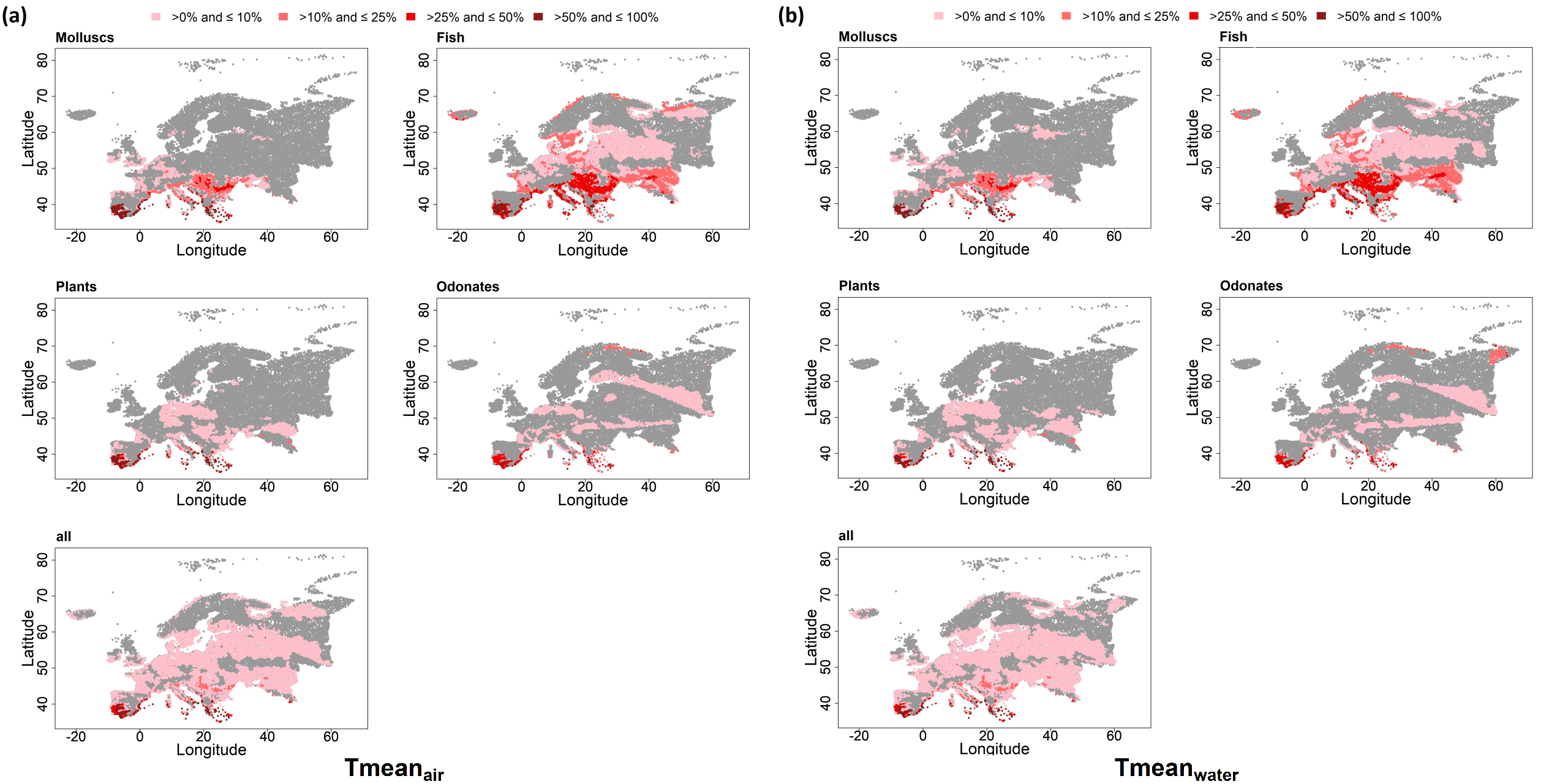
**

**Fig. S1.15** Comparison of the relative frequency per catchment of species with the critical maximum temperature (CT) inferred from (a) Tmean_air_ and (b) Tmean_water_ that is exceeded by the averaged projected temperature of the three climate models MOHC, IPSL and MPI for the 2050s. The grey area represents either no occurrence or catchments in which the CT, i.e. the maximum temperature of a species’ occurrence, is not exceeded by the projected temperatures. Note that crayfish were excluded because of the low frequency of analysed species.


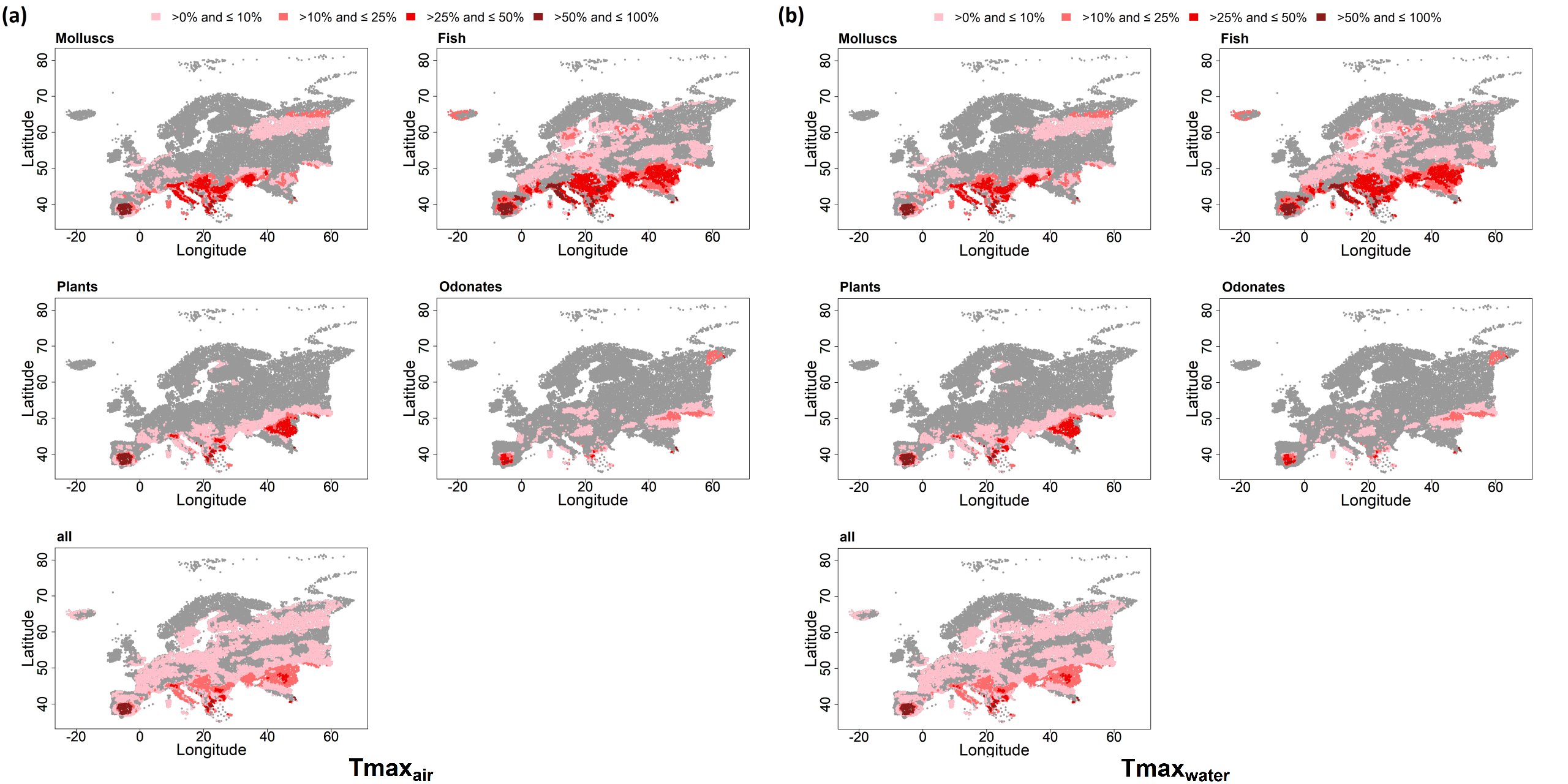


**Fig. S1.16** Comparison of the relative frequency per catchment of species with the critical maximum temperature (CT) inferred from (a) Tmax_air_ and (b) Tmax_water_ that is exceeded by the averaged projected temperature of the three climate models MOHC, IPSL and MPI for the 2050s. The grey area represents either no occurrence or catchments in which the CT, i.e. the maximum temperature of a species’ occurrence, is not exceeded by the projected temperatures. Note that crayfish were excluded because of the low frequency of analysed species.
